# Evaluation of Endothelial Dysfunction and Inflammatory Vasculopathy After SARS-CoV-2 Infection—A Cross-Sectional Study

**DOI:** 10.3389/fcvm.2021.750887

**Published:** 2021-10-13

**Authors:** Philipp Jud, Paul Gressenberger, Viktoria Muster, Alexander Avian, Andreas Meinitzer, Heimo Strohmaier, Harald Sourij, Reinhard B. Raggam, Martin Helmut Stradner, Ulrike Demel, Harald H. Kessler, Kathrin Eller, Marianne Brodmann

**Affiliations:** ^1^Division of Angiology, Department of Internal Medicine, Medical University of Graz, Graz, Austria; ^2^Institute for Medical Informatics, Statistics and Documentation, Medical University of Graz, Graz, Austria; ^3^Clinical Institute of Medical and Chemical Laboratory Diagnostics, Medical University of Graz, Graz, Austria; ^4^Department Center of Medical Research, Medical University of Graz, Graz, Austria; ^5^Division of Endocrinology, Department of Internal Medicine, Medical University of Graz, Graz, Austria; ^6^Division of Rheumatology and Immunology, Department of Internal Medicine, Medical University of Graz, Graz, Austria; ^7^Diagnostic and Research Institute of Hygiene, Microbiology and Environmental, Medical University of Graz, Graz, Austria; ^8^Division of Nephrology, Department of Internal Medicine, Medical University of Graz, Graz, Austria

**Keywords:** COVID-19, endothelial dysfunction, inflammation, vasculopathy, capillary changes

## Abstract

**Background:** Rising data suggest that COVID-19 affects vascular endothelium while the underlying mechanisms promoting COVID-19-associated endothelial dysfunction and inflammatory vasculopathy are largely unknown. The aim was to evaluate the contribution of COVID-19 to persisting vascular injury and to identify parameters linked to COVID-19-associated endothelial dysfunction and inflammatory vasculopathy.

**Methods:** In a cross-sectional design, flow-mediated dilation (FMD), nitroglycerine-related dilation (NMD), pulse-wave velocity (PWV), augmentation index, intima-media thickness (IMT), compounds of the arginine and kynurenine metabolism, homocysteine, von Willebrand factor (vWF), endothelial microparticles (EMP), antiendothelial cell antibodies, inflammatory, and immunological parameters, as well as nailfold capillary morphology were measured in post-COVID-19 patients, patients with atherosclerotic cardiovascular diseases (ASCVD) and healthy controls without prior or recent SARS-CoV-2 infection.

**Results:** Post-COVID-19 patients had higher values of PWV, augmentation index, IMT, asymmetric and symmetric dimethylarginine, vWF, homocysteine, CD31+/CD42b– EMP, C-reactive protein, erythrocyte sedimentation rate, interleukin-6, and β-2-glycoprotein antibodies as well as lower levels of homoarginine and tryptophan compared to healthy controls (all with *p* < 0.05). A higher total number of pathologically altered inflammatory conditions and higher rates of capillary ramifications, loss, caliber variability, elongations and bushy capillaries with an overall higher microangiopathy evolution score were also observed in post-COVID-19 patients (all with *p* < 0.05). Most parameters of endothelial dysfunction and inflammation were comparably altered in post-COVID-19 patients and patients with ASCVD, including FMD and NMD.

**Conclusion:** COVID-19 may affect arterial stiffness, capillary morphology, EMP and selected parameters of arginine, kynurenine and homocysteine metabolism as well as of inflammation contributing to COVID-19-associated endothelial dysfunction and inflammatory vasculopathy.

## Introduction

COVID-19 caused by the severe acute respiratory syndrome coronavirus 2 (SARS-CoV-2) has evolved into a pandemic since it was detected in the end of 2019. A higher mortality rate was reported among patients with preexisting cardiovascular diseases compared to patients without an underlying disease and cardiovascular diseases seem to be risk factors for severe SARS-CoV-2 infection (1–3). Additionally, there are rising data suggesting that SARS-CoV-2 affects directly and indirectly endothelial cells, thus leading to endothelial injury and dysfunction thereby contributing to thromboembolism, vasculitic changes and abnormal nailfold capillaroscopy (4–9). The underlying mechanisms promoting COVID-19-associated endothelial dysfunction and inflammatory vasculopathy are yet still largely unknown while potential dysregulation of the renin-angiotensin-aldosterone system, immunothrombosis, and direct endothelial infection have been proposed (10–12).

Endothelial dysfunction may be a key contributor of vasculopathy due to underlying functional and structural changes of endothelial cells, and numerous parameters have been attributed to endothelial dysfunction. Flow-mediated dilation (FMD), pulse-wave velocity (PWV), and intima-media thickness (IMT) represent widely used, non-invasive indicators of vascular reactivity, arterial stiffness, and morphological changes of large arteries (13–15). All have been thoroughly evaluated in atherosclerotic cardiovascular diseases (ASCVD) as predictors for cardiovascular events and mortality (16–18). Additionally, homocysteine, kynurenine and compounds of the arginine metabolism, like homoarginine, asymmetric dimethylarginine (ADMA) and symmetric dimethylarginine (SDMA), are important mediators of endothelial dysfunction representing further predictors of cardiovascular mortality (19–22). Moreover, endothelial microparticles (EMP), which are released during apoptosis or activation of endothelial cells, as well as antiendothelial cell antibodies (AECA) may be associated with endothelial injury and activation, thus contributing to vasculopathy and endothelial dysfunction (23, 24).

Data about the respective parameters of endothelial dysfunction and inflammatory vasculopathy are largely lacking in COVID-19. Furthermore, data investigating if SARS-CoV-2 infection may cause persistent endothelial and vascular immunopathologic changes are also very limited. The aim of this study was to investigate if previous SARS-CoV-2 infection contributes to persisting endothelial dysfunction, inflammatory vasculopathy, macro-, and microvascular changes and to compare these findings to patients with ASCVD and healthy controls without SARS-CoV-2 infection.

## Materials and Methods

### Study Population and Design

**Figure 1:**
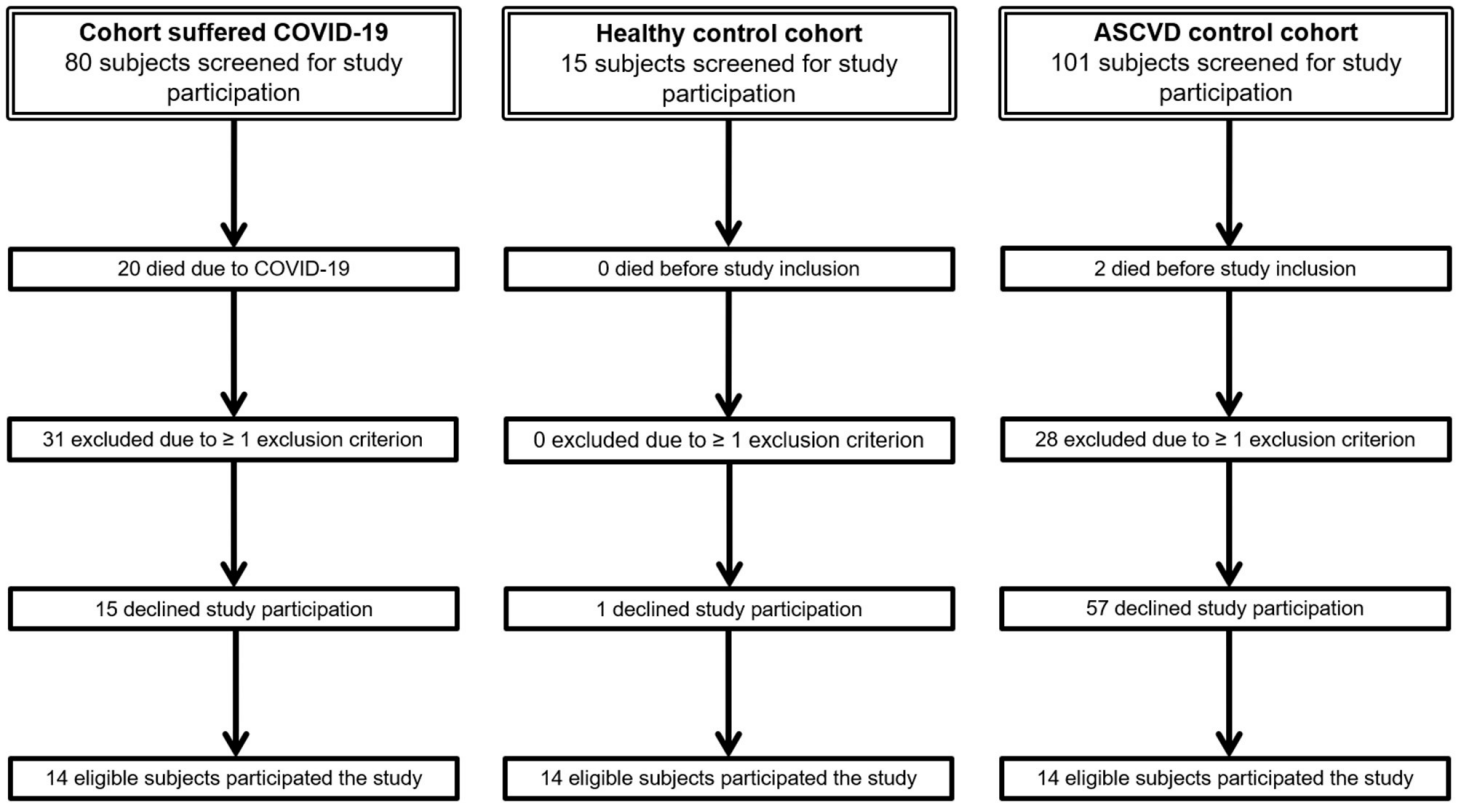
Flow chart of study recruitment.

Post-COVID-19 patients diagnosed between March and April 2020 and inpatient treatment at the division of Angiology of the Medical University of Graz were screened *via* charts review for study inclusion and invited to participate. For every COVID-19 subject, one sex-matched healthy volunteer was recruited as well as one age—(± 1 year) and sex-matched subject with known ASCVD was also screened for study inclusion and invited to participate in the study (Figure 1). Overall, 42 subjects participated that study which were subdivided into three respective groups with 14 subjects per group. Inclusion criterion for the group of patients with COVID-19 was a known prior SARS-CoV-2 infection. Inclusion criterion for the ASCVD group was the presence of at least one detected, asymptomatic or symptomatic ASCVD, either coronary artery disease, or cerebrovascular disease, or lower extremity arterial disease (LEAD) or upper extremity arterial disease (UEAD). Exclusion criteria for all three cohorts were age < 18 years, any type of preexisting connective tissues disease or vasculitis, existing autoimmune diseases, recent pregnancy, recent malignancies and any acute infections, including foot ulcers or necrosis, at time of enrollment. For the group of COVID-19 subjects, preexisting history of diabetes mellitus, asymptomatic and symptomatic ASCVD, including angina pectoris, myocardial infarction, stroke, intermittent claudication, rest pain, and/or necrosis or ulcers of the lower or upper extremity, were additional exclusion criteria. All subjects were instructed to withhold potentially vasodilatory medications, including calcium channel blockers, phosphodiesterase-5 inhibitors, or prostanoids, and anticoagulation at least 24 h prior to study measurements. All participating patients with ASCVD and healthy controls underwent measurement of COVID-19 immunoglobulin (Ig) G antibodies and detection of SARS-CoV-2 RNA by polymerase chain reaction (PCR) testing within 3 days prior to start of the study in order to exclude a preexisting or recent SARS-CoV-2 infection. SARS-CoV-2 IgG antibodies were measured by the LIAISON^®^ SARS-CoV-2 S1/S2 IgG (DiaSorin, Saluggia, Italy). This fully automated test allows detection and quantitation of IgG antibodies against S1/S2 antigens of SARS-CoV-2. For detection of SARS-CoV-2 RNA, oropharyngeal swabs were collected by using the Copan ESwab collection system containing 1 ml of transport medium. Samples were tested for SARS-CoV-2 RNA at the Molecular Diagnostics Laboratory, Medical University of Graz, within 12 h of arrival. Presence of SARS-CoV-2 RNA was determined by real-time PCR using the SARS-CoV-2 Test for use on the cobas^®^ 6800/8800 Systems (Roche Molecular Diagnostics, Pleasanton, USA). With this assay, selective amplification of target nucleic acid from the sample is achieved by the use of target-specific forward and reverse primers for ORF1a/b nonstructural region that is unique to SARS-CoV-2. In addition, a conserved region in the structural protein envelope E-gene is chosen for pan-Sarbecovirus detection. The pan-Sarbecovirus detection set also detects SARS-CoV-2 virus. No study subject had received COVID-19 vaccines prior to study measurements.

Between September 2020 and March 2021, parameters of endothelial dysfunction, immune-inflammatory parameters, and capillary morphology of the nailfold were investigated. Primary endpoint was the difference of FMD between post-COVID-19 patients, patients with ASCVD and healthy controls. Secondary endpoints were differences of nitroglycerine-related dilation (NMD), PWV, IMT, homocysteine, compounds of the arginine metabolism, kynurenine, tryptophan, von Willebrand factor (vWF), EMP, AECA, immune-inflammatory parameters and capillary morphology of the nailfold between patients with previous SARS-CoV-2 infection, ASCVD and healthy controls. After signing the informed consent form, blood sampling or biochemical analysis were obtained followed by medical history evaluating cardiovascular risk factors. Subsequently, pulse-wave analysis and measurements of IMT, FMD, NMD and capillary changes by nailfold video capillaroscopy (NVC) were performed. Measurements of pulse-wave analysis, IMT, FMD, NMD, laboratory parameters, and NVC were performed in the morning between 7:00 a.m. and 9:00 a.m. after an overnight fast in a temperature-controlled (22–24°C) and quiet room.

### Pulse-Wave Analysis, Intima-Media Thickness, and Flow-Mediated Dilation

Pulse-wave analysis including aortic PWV, augmentation index and pulse pressure was measured and calculated *via* the oscillometric device Mobil-O-Graph^®^ (I.E.M., Aachen, Germany) by automated analysis. After obtainment of blood samples and a rest of 5 min, size-adjusted cuff was placed on the right upper arm about 2–4 cm above the ante-cubital fossa in supine position and subsequent pulse-wave analysis was performed. The patients were requested not to speak and not to move over the whole pulse-wave analysis. PWV of >10 m/s was defined as pathologic PWV (17).

Measurement of the IMT of the common carotid, axillary and superficial femoral artery was assessed in supinely positioned patients. After further rest of 5 min, both common carotid, axillary, and femoral arteries were examined in a longitudinal plane using a high-resolution linear array probe with 8–13 MHz (Siemens ACUSON S2000™, Siemens Healthcare Corp., Erlangen, Germany). The thickness of the intimal and medial layers of the vascular wall was measured on frozen longitudinal images in at least 1-cm-long segment of the artery. Three IMT measurements were performed per subject and per anatomic location while the mean value of the three measurements of the respective location was recorded.

All FMD measurements were performed by the same trained technician according to recent guidelines (13). All recommendations of those guidelines were fulfilled regarding subject preparation, protocol, and operator-dependent factors while sublingual administration of 0.4 mg glyceryl trinitrate was used instead of recommended 25 μg glyceryl trinitrate. Guideline recommendations for technique and analysis, including continuous measurement of velocity and diameter using simultaneous live duplex ultrasound and the use of continuous edge-detection and wall tracking software calculating peak diameter and shear rate stimulus, could not be fulfilled since such a software was not available during the study. Instead, offline analysis by a blinded observer was performed. A blood pressure cuff was placed below the antecubital fossa on the forearm and the baseline diameter of brachial artery was examined in a longitudinal plane between 2 and 7 cm proximal to the antecubital fossa. Three end-diastolic diameters between two intimal layers were measured ECG-gated during image acquisition in a one-centimeter-long segment of the brachial artery. Afterwards, the cuff was inflated >50 mmHg above the resting systolic pressure for 5 min and then deflated. The postischemic diameter of the brachial artery was measured 60 s after cuff release. FMD was defined as the change in postischemic diameter as a percentage of the baseline diameter. After a rest of 15 min, NMD was performed. Diameter of the brachial artery was recorded similar to the technique described for FMD before and 5 min after sublingual administration of 0.4 mg glyceryl trinitrate spray. All FMD and NMD measurements were performed using a conventional ultrasound scanner (Siemens ACUSON S2000™, Siemens Healthcare Corp., Erlangen, Germany) with an 8–13 MHz linear array transducer. Additionally, values of FMD < 7% and of NMD < 15.6% were defined as pathologic FMD and NMD values according to proposed reference values (25, 26).

### Biochemical Analyses

Fasting blood samples for evaluation of L-arginine, homoarginine, citrulline, ornithine, ADMA, and SDMA, kynurenine, tryptophan, vWF, homocysteine, AECA, and EMP and immune-inflammatory parameters were obtained. Present leukocytosis, lymphopenia, hypocomplementemia, elevated levels of C-reactive protein (CRP), erythrocyte sedimentation rate (ESR), serum amyloid A (SAA), interleukin 6 (IL-6), antinuclear antibodies (ANA), extractable nuclear antigen (ENA) antibodies, antiphospholipid antibodies, anti-neutrophil cytoplasmic antibodies (ANCA), anti-citrullinated protein (ACP) antibodies, rheumatoid factor, and cytoplasmic antibodies, as well as decreased and increased levels of Ig were additionally recorded. Detailed list of the respective immune-inflammatory parameters is shown in Table 1.

**Table 1:** Immune-inflammatory parameters and cut-off values indicating potentially inflammatory conditions.

White blood cells (WBC)	Antinuclear antibodies (ANA)
C-reactive protein (CRP)	Extractable nuclear antigen (ENA) antibodies
Erythrocyte sedimentation rate (ESR)	Lupus anticoagulant
Serum amyloid A (SAA)	Cardiolipin and β-2-glycoprotein antibodies
Complement factors C3 and C4	Anti-neutrophil cytoplasmic antibodies (ANCA)
Interleukin 6 (IL-6)	Anti-citrullinated protein (ACP) antibodies
Immunoglobulin (Ig) A, G, M	Rheumatoid factor
IgG subclasses 1–4	Cytoplasmic antibodies
**Definitions of pathologically altered inflammatory conditions**	
Leucocytosis > 11.3 × 10^9^/L	Elevated antiphospholipid antibodies: Lupus anticoagulant > 45 s
Lymphopenia < 20%	Cardiolipin antibodies > 10 U/mL
Elevated CRP > 5 mg/L	β-2-glycoprotein antibodies > 10 U/mL
Elevated ESR > 20 mm/h	Elevated ANCA: MPO-ANCA > 5 U/mL
Elevated SAA > 6.4 mg/L	PR3-ANCA > 10 U/mL
Hypocomplementemia	c-ANCA ≥ 1:80
C3 < 0.9 g/L	p-ANCA titer ≥ 1:80
C4 < 0.1 g/L	x-ANCA ≥ 1:80
Elevated IL-6 > 7.0 pg/mL	Elevated ACP antibodies > 10 U/mL
Elevated ANA titer ≥ 1:80	Elevated rheumatoid factor > 20 U/mL
Elevated ENA antibodies > 1 U/mL	Positive cytoplasmic antibodies
Decreased Ig	Elevated Ig
IgA < 0.7 g/L	IgA > 4 g/L
IgG < 7 g/L	IgG > 16 g/L
IgM < 0.4 g/L	IgM > 2.3 g/L
IgG_1_ < 4.05 g/L	IgG_1_ > 10.11 g/L
IgG_2_ < 1.69 g/L	IgG_2_ > 7.86 g/L
IgG_3_ < 0.11 g/L	IgG_3_ > 0.85 g/L
IgG_4_ < 0.03 g/L	IgG_4_ > 2.01 g/L

Blood sample for measurement of parameters of the arginine and kynurenine metabolism as well as AECA were centrifuged at 4,000 × g for 10 min at 15°C temperature within 1 h after blood sampling obtainment. The supernatant was collected and divided into aliquots of 1 ml, which were stored at −80°C until final analysis. Amino acids and metabolites were measured by high-performance liquid chromatography as described elsewhere (27–29). L-arginine/ADMA, L-arginine/SDMA, homoarginine/ADMA, homoarginine/SDMA, L-arginine/ornithine, citrulline/L-arginine, citrulline/ornithine, global arginine bioavailability (GAB) ratio, defined as ratio of L-arginine over ornithine plus citrulline, and kynurenine/tryptophan were calculated by division of the respective parameter. AECA were measured by enzyme-linked immunosorbent assay (ELISA) method using a qualitative ELISA kit (Cusabio Technology, Wuhan, China) according to the user manual.

EMP were measured according to the recommendations for the analysis of extracellular vesicles published by Cossarizza et al. (30). Blood samples were collected in 5 ml citrate tubes after discarding the first 2 ml of blood without venous stasis and kept in upright position. Within 1 h after obtaining blood sampling, the plasma was centrifuged at 2,500 × g for 15 min at room temperature to obtain platelet-poor plasma. One milliliter of the supernatant was centrifuged again at 2,500 × g for 15 min at room temperature to obtain platelet-free plasma. The supernatant was collected and divided into aliquots of 0.1 ml, which were snap-frozen in liquid nitrogen and stored at −80°C until further analysis. A platelet-free plasma aliquot was thawed in a water bath at 37°C and immediately processed for fluorescence staining. Twenty-five microliters of platelet-free plasma was mixed with fluorochrome-labeled anti-human CD31, CD42b, CD51, CD54, CD62E, CD105, and CD144 antibodies (Biolegend, San Diego, USA) and incubated for 1.5 h at 4°C in the dark, followed by incubation with fluorescein-isothiocyanate-labeled lactadherin (CellSystems, Troisdorf, Germany) for another 30 min. Lactadherin binds specifically to phosphatidylserine on the outer surface of extracellular vesicles. Corresponding fluorochrome-labeled isotype antibodies were used as negative controls. After incubation, the samples were diluted 1:50 with 0.22 μm filtered phosphate buffered saline prior to flow cytometric analysis. EMP were identified as events that are positive for the above-mentioned markers and negative for CD42b. CD42b was used to distinguish EMP from platelet-derived microparticles (31). A microparticle gate was established using fluorescent 1 μm silica beads (Kisker Biotech, Steinfurt, Germany) for size calibration.

The remaining laboratory parameters were measured in sera and plasma samples of the patients at a single central lab of the Medical University of Graz.

### Nailfold Video Capillaroscopy and Capillary Changes

NVC of the second to the fifth finger on both hands was performed in sitting position after pulse-wave analysis (Skinview, Optometron Ltd., Ismaning, Germany). Morphological changes of the capillaries, including microhemorrhages, capillary edema, capillary ramifications, bushy capillaries, capillary loss, giant capillaries, capillary ectasia, tortuous capillaries, capillary caliber variability, elongated capillaries, capillary thrombosis and disorganization of the microvascular array were recorded and a semi-quantitative rating scale to score each capillary abnormality was adopted (0 = no changes; 1 = <33% of capillary changes; 2 = 33–66% of capillary changes; 3 = more than 66% of capillary changes, per linear millimeter). The score values from the eight digits were added together and divided by eight resulting in the final score values. Microvascular disease activity was assessed by capillaroscopic skin ulcer risk index (CSURI) and microangiopathy evolution score (MES) (32, 33). Microvascular changes were also quantified into early, active and late pattern, as defined by Cutolo et al. (34).

### Statistical Analysis

Categorical variables were represented by frequency and percentages. Continuous variables were given as median and interquartile range or as mean ± standard deviation (SD). Normal distribution was examined *via* Shapiro–Wilk test. In case of normally distributed data, two-sided *t*-test was used and for non-normally distributed data Mann–Whitney *U*-test was utilized. *P* < 0.05 were assumed as statistically significant and statistical analyses were executed *via* SPSS version 26.0.

### Ethical Approval

The study was approved by the Institutional Review Board of the Medical University Graz, Austria (EK 32-502 ex 19/20). All patients gave their written informed consent.

## Results

Fourteen post-COVID-19 patients (7 male, 50%) with a mean age (± SD) of 68.7 (±12.0) years, 14 sex-matched healthy controls with a mean age (± SD) of 30.7 (±4.2) years, and 14 sex- and age-matched patients with ASCVD and a mean age (± SD) of 66.9 (±10.9) years participated in that study. Age-matching was impossible for two patients with ASCVD due to a high refusal rate of study participation (Figure 1). No subject of the healthy controls and ASCVD controls had a positive COVID-19 PCR or COVID-19 antibody testing. Patients characteristics are shown in Table 2.

**Table 2:** Patients' characteristics.

	**COVID-19 (*n* = 14)**	**ASCVD (*n* = 14)**	**Controls (*n* = 14)**
**Patients**, ***n*** **(%)**			
Female	7 (50.0%)	7 (50.0%)	7 (50.0%)
Male	7 (50.0%)	7 (50.0%)	7 (50.0%)
**Age (years), mean (± SD)**	68.7 ± 12.0^*^	66.9 ± 10.9^†^	30.7 ± 4.2
**Duration after SARS-CoV-2 infection (weeks), mean (± SD)**	28.6 ± 3.0	–	–
**COVID-19 phenotype**, ***n*** **(%)**			
COVID-19 pneumonia	14 (100.0)	–	–
COVID-19 ARDS	3 (21.4)	–	–
**Disease duration of ASCVD (weeks), median (25th−75th percentile)**	–	293.3 (62.1–529.9)	–
**Prior familial ASCVD**, ***n*** **(%)**	5 (35.7)	9 (64.3)	4 (28.6)
**BMI (kg/m** ^**2**^ **), mean (± SD)**	29.4 ± 8.3^*^	27.6 ± 4.5^†^	23.8 ± 3.2
**HbA**_**1c**_ **(mmol/mol), median (25th−75th percentile)**	39 (33–42)^*^	41 (37–47)^†^	33 (32–34)
**eGFR (ml/min/1.73 m** ^2^ **), median (25th−75th percentile)**	84.4 (72.2–90.7)^*^	76.7 (65.1–89.1)^†^	103.4 (97.6–115.7)
**Current sport activity**, ***n*** **(%)**	8 (57.1)^*^	5 (35.7)^†^	13 (92.9)
Times per week (*n*), median (25th−75th percentile)	2 (0–3)^*^	0 (0–3)^†^	4 (2–5)
Duration per week (min), median (25th−75th percentile)	30 (0–60)^‡^	0 (0–45)^†^	50 (30–60)
**Previous history**, ***n*** **(%)**			
COPD	1 (7.1%)	3 (21.4)	0 (0.0)
**Smoking**			
*Current*	0 (0.0)^*^^‡^	5 (35.7)	4 (28.6)
*Ex*	6 (42.9)	6 (42.9)	4 (28.6)
*Non-smokers*	8 (57.1)	3 (21.4)	6 (42.9)
Bronchial asthma	0 (0.0)	0 (0.0)	0 (0.0)
Arterial hypertension	6 (42.9)^*^^‡^	13 (92.9)^†^	0 (0.0)
Diabetes mellitus	0 (0.0)^‡^	4 (28.6)^†^	0 (0.0)
Atrial fibrillation	1 (7.1)	2 (14.3)	0 (0.0)
Hypercholesterolemia	6 (42.9)^*^^‡^	12 (85.7)^†^	0 (0.0)
Hypertriglyceridemia	2 (14.3)^‡^	7 (50.0)^†^	0 (0.0)
CKD	1 (7.1)	3 (21.4)	0 (0.0)
Inactive malignancy	4 (28.6)^*^	1 (7.1)	0 (0.0)
Coronary artery disease	0 (0.0)^‡^	8 (57.1)^†^	0 (0.0)
*Myocardial infarction*	0 (0.0)^‡^	4 (28.6)^†^	0 (0.0)
Cerebrovascular disease	0 (0.0)^‡^	11 (78.6)^†^	0 (0.0)
*Stroke*	0 (0.0)	2 (14.3)	0 (0.0)
Upper extremity arterial disease	0 (0.0)	3 (21.4)	0 (0.0)
Lower extremity arterial disease	0 (0.0)^‡^	13 (92.9)^†^	0 (0.0)
Renal artery disease	0 (0.0)	1 (7.1)	0 (0.0)
Mesenteric artery disease	0 (0.0)	1 (7.1)	0 (0.0)
PCI/PTA	0 (0.0)^‡^	11 (78.6)^†^	0 (0.0)
**Drug therapy**, ***n*** **(%)**			
ACE inhibitors	3 (21.4)	4 (28.6)^†^	0 (0.0)
ARB	2 (14.3)	5 (35.7)^†^	0 (0.0)
Beta blockers	3 (21.4)^‡^	9 (64.3)^†^	0 (0.0)
Calcium antagonists	2 (14.3)	5 (35.7)^†^	0 (0.0)
Diuretics	1 (7.1)	4 (28.6)^†^	0 (0.0)
Other antihypertensives	0 (0.0)	0 (0.0)	0 (0.0)
Antiplatelet therapy	0 (0.0)^‡^	9 (64.3)^†^	0 (0.0)
Oral anticoagulation	2 (14.3)	3 (21.4)	0 (0.0)
Statins	0 (0.0)^‡^	10 (71.4)^†^	0 (0.0)
PCSK-9 inhibitors	0 (0.0)	2 (14.3)	0 (0.0)
Metformin	0 (0.0)^‡^	4 (28.6)^†^	0 (0.0)
Other oral antihyperglycemic agents	0 (0.0)	2 (14.3)	0 (0.0)
Insulin	0 (0.0)	0 (0.0)	0 (0.0)

*ACE, angiotensin-converting enzyme; ARB, Angiotensin receptor blockers; ARDS, acute respiratory distress syndrome; ASCVD, atherosclerotic cardiovascular diseases; BMI, body mass index; CKD, chronic kidney disease; COPD, chronic obstructive pulmonary disease; eGFR, estimated glomerular filtration rate; PCI, percutaneous coronary intervention; PCSK-9, proprotein convertase subtilisin/kexin type 9; PTA, percutaneous transluminal angioplasty*.

* *< 0.05 between group with previous COVID-19 and healthy controls*.

† *p < 0.05 between group with ASCVD and healthy controls*.

‡ *p < 0.05 between group with previous COVID-19 and group with ASCVD*.

### Endothelial Dysfunction and Macrovascular Changes

No difference between all three groups were found for FMD and NMD. Post-COVID-19 patients had a higher rate of pathologic aortic PWV with >10 m/s (*p* = 0.001) and higher values of aortic PWV, augmentation index, IMT of the common carotid, axillary and superficial femoral artery, ADMA, SDMA, kynurenine/tryptophan ratio, vWF antigen and activity, homocysteine and CD31+/CD42b– EMP compared to healthy controls (*p* < 0.001; *p* = 0.009; *p* < 0.001; *p* < 0.001; *p* < 0.001; *p* = 0.001; *p* = 0.043; *p* = 0.001; *p* = 0.002; *p* = 0.004; *p* = 0.004; *p* = 0.020, respectively). In the group of post-COVID-19 patients, values of those respective parameters were comparable to patients with ASCVD without significant differences, except for IMT of the axillary artery, which was lower (*p* = 0.017), and for CD31+/CD42b– EMP, which were higher (*p* = 0.012) in the COVID-19 group. Significantly lower values of homoarginine, tryptophan, L-arginine/ADMA, homoarginine/ADMA, and homoarginine/SDMA ratio were found in post-COVID-19 patients compared to healthy controls (*p* = 0.004; *p* = 0.027; *p* < 0.001; *p* < 0.001; *p* = 0.002, respectively), which were again comparable to the values of patients with established ASCVD. Ornithine was lower and L-arginine/ornithine and GAB ratio were higher in post-COVID-19 patients compared to patients with ASCVD (*p* = 0.001; *p* = 0.020; *p* = 0.022, respectively). AECA, CD54+/CD42b–, CD62E+/CD42b–, CD105+/CD42b–, and CD144+/CD42b– EMP were undetectable in all three groups (Table 3).

**Table 3:** Parameters of endothelial dysfunction.

	**COVID-19 (*n* = 14)**	**ASCVD (*n* = 14)**	**Controls (*n* = 14)**
FMD (%), mean (± SD)	4.44 ± 2.90	3.17 ± 2.95	4.58 ± 3.48
<7%, *n* (%)	10 (71.4%)	11 (78.6%)	10 (71.4%)
NMD (%), mean (± SD)	16.78 ± 6.32	17.11 ± 9.23	20.60 ± 8.46
<15.6%, *n* (%)	5 (38.5%)	7 (50.0%)	3 (21.4%)
Aortic PWV (m/s), median (25th−75th percentile)	10.75 (8.10–11.45)^*^	9.95 (8.40–11.60)^†^	5.70 (5.38–6.05)
>10m/s, *n* (%)	8 (57.1%)^*^	7 (50.0%)^†^	0 (0.0%)
Augmentation index (%), median (25th−75th percentile)	22 (10–40)^*^	33 (24–39)^†^	4 (1–11)
Pulse pressure (mmHg), median (25th−75th percentile)	47 (35–50)	52 (48–67)	49 (41–53)
**IMT (mm), median (25th−75th percentile)**			
IMT common carotid artery average	0.59 (0.52–0.68)^*^	0.72 (0.60–1.01)^†^	0.44 (0.40–0.45)
IMT axillary artery average	0.58 (0.45–0.64)^*^^‡^	0.71 (0.59–0.88)^†^	0.40 (0.39–0.46)
IMT superficial femoral artery average	0.54 (0.47–0.62)^*^	0.55 (0.43–0.61)^†^	0.40 (0.36–0.40)
ADMA (μmol/L), median (25th−75th percentile)	0.76 (0.65–0.79)^*^	0.80 (0.72–0.83)^†^	0.60 (0.62–0.65)
SDMA (μmol/L), median (25th−75th percentile)	0.73 (0.65–0.86)^*^	0.84 (0.65–1.07)^†^	0.65 (0.62–0.70)
L-arginine (μmol/L), median (25th−75th percentile)	119.74 (113.40–142.29)	136.64 (120.34–149.80)	132.50 (107.62–143.44)
Homoarginine (μmol/L), median (25th−75th percentile)	1.59 (1.16–2.31)^*^	1.59 (1.41–2.21)^†^	2.36 (1.90–3.45)
Citrulline (μmol/L), median (25th−75th percentile)	34.59 (31.13–38.69)	39.21 (32.46–50.94)	32.11 (25.91–40.24)
Ornithine (μmol/L), median (25th−75th percentile)	66.17 (63.33–73.63)^‡^	94.30 (80.98–114.48)^†^	64.25 (38.44–78.30)
L-arginine/ADMA ratio, median (25th−75th percentile)	173.33 (143.28–188.47)^*^	165.88 (151.23–192.94)^†^	207.22 (200.43–224.48)
L-arginine/SDMA ratio, median (25th−75th percentile)	167.33 (132.87–188.57)	153.82 (125.35–201.30)^†^	190.40 (167.97–220.01)
Homoarginine/ADMA ratio, median (25th−75th percentile)	2.16 (1.47–2.90)^*^	2.02 (1.75–2.82)^†^	3.75 (2.99–5.67)
Homoarginine/SDMA ratio, median (25th−75th percentile)	2.01 (1.39–3.20)^*^	2.04 (1.38–2.93)^†^	3.79 (2.89–5.19)
L-arginine/ornithine ratio, median (25th−75th percentile)	1.88 (1.53–2.11)^‡^	1.49 (1.10–1.78)^†^	2.22 (1.46–2.82)
Citrulline/L-arginine ratio, median (25th−75th percentile)	0.28 (0.21–0.31)	0.31 (0.22–0.38)	0.27 (0.22–0.28)
Citrulline/ornithine ratio, median (25th−75th percentile)	0.50 (0.39–0.56)	0.43 (0.40–0.46)	0.59 (0.39–0.66)
GAB ratio, median (25th−75th percentile)	1.20 (1.06–1.42)^‡^	0.95 (0.77–1.17)^†^	1.38 (1.07–1.71)
Kynurenine (μmol/L), median (25th−75th percentile)	2.45 (2.00–3.14)	2.96 (2.37–3.23)^†^	2.21 (2.00–2.39)
Tryptophan (μmol/L), median (25th−75th percentile)	54.40 (49.97–59.15)^*^	59.52 (54.33–66.96)	61.76 (56.25–70.70)
Kynurenine/tryptophan ratio, median (25th−75th percentile)	0.050 (0.040–0.053)^*^	0.045 (0.040–0.050)^†^	0.030 (0.030–0.040)
vWF antige *n* (%), mean (± SD)	138.6 ± 14.1^*^	137.8 ± 11.1^†^	109.3 ± 25.3
vWF activity (%), mean (± SD)	168.5 ± 60.8^*^	177.9 ± 55.3^†^	110.5 ± 31.5
Homocysteine (μmol/L), median (25th−75th percentile)	12.3 (10.5–14.8)^*^	9.7 (6.6–14.8)	9.0 (8.6–10.4)
**AECA**, ***n*** **(%)**			
Positive	0 (0.0)	0 (0.0)	0 (0.0)
Negative	14 (100.0)	14 (100.0)	14 (100.0)
**EMP (U/μl)**			
CD31+/CD42b–	201.25 (158.88–279.50)^*^‡	115.50 (90.88–169.75)	137.50 (73.00–171.38)
CD51+/CD42b–	13.50 (5.25–49.25)	27.75 (19.38–39.63)	22.25 (17.88–28.50)
CD54+/CD42b–	–^§^	–^§^	–^§^
CD62E+/CD42b–	–^§^	–^§^	–^§^
CD105+/CD42b–	–^§^	–^§^	–^§^
CD144+/CD42b–	–^§^	–^§^	–^§^

*ADMA, asymmetric dimethylarginine; AECA, antiendothelial cell antibodies; ASCVD, atherosclerotic cardiovascular diseases; EMP, endothelial microparticles; FMD, flow-mediated dilation; GAB, global arginine bioavailability; IMT, intima-media thickness; NMD, nitroglycerine-related dilation; PWV, pulse-wave velocity; SDMA, symmetric dimethylarginine; vWF, von Willebrand factor*.

* *p < 0.05 between group with previous COVID-19 and healthy controls*.

† *p <0.05 between group with ASCVD and healthy controls*.

‡ *p <0.05 between group with previous COVID-19 and group with ASCVD*.

§ *not detectable*.

### Inflammation

Higher values of CRP, ESR, IL-6, and β-2-glycoprotein antibodies as well as higher frequencies of CRP elevation and any Ig decrease were observed for post-COVID-19 patients compared to healthy controls (*p* = 0.009; *p* = 0.007; *p* = 0.004; *p* = 0.031; *p* = 0.007; *p* = 0.015, respectively). Again, these parameters were comparable between the ASCVD und the COVID-19 cohort, without statistically significant differences. Post-COVID-19 patients revealed higher levels of PR3-ANCA, IgM, and IgG_2_ compared to patients with ASCVD (*p* = 0.016; *p* = 0.011; *p* = 0.036, respectively), but not to healthy controls. Healthy controls had lower levels of C3 and C4 and higher rates of hypocomplementemia than patients with previous COVID-19 and with ASCVD (all with *p* < 0.05). Post-COVID-19 patients had a higher total number of pathologically altered inflammatory conditions compared to healthy controls (*p* = 0.016), but not to patients with ASCVD (*p* = 0.385 (Table 4).

**Table 4:** Immune-inflammatory parameters.

	**COVID-19 (*n* = 14)**	**ASCVD (*n* = 14)**	**Controls (*n* = 14)**
WBC (10^9^/L), median (25th−75th percentile)	6.0 (5.7–6.6)	6.8 (5.1–8.2)	5.3 (3.9–7.0)
Leukocytosis, *n* (%)	0 (0.0%)	0 (0.0%)	0 (0.0%)
Lymphocytes (10^9^/L), median (25th−75th percentile)	1.5 (1.1–1.7)	1.5 (1.3–2.2)	1.6 (1.2–1.9)
Lymphopenia, *n* (%)	3 (21.4%)	5 (35.7%)	1 (7.1%)
CRP (mg/dL), median (25th−75th percentile)	2.5 (0.8–7.8)^*^	1.9 (0.8–3.8)^†^	0.7 (0.5–1.3)
CRP elevation, *n* (%)	6 (42.9%)^*^	3 (21.4%)	0 (0.0%)
ESR (mm/h), median (25th−75th percentile)	7 (5–10)^*^	9 (4–15)^†^	2 (2–5)
ESR elevation > 20, *n* (%)	1 (7.1%)	0 (0.0%)	0 (0.0%)
SAA (mg/L), median (25th−75th percentile)	4.5 (2.2–9.3)	5.7 (3.3–8.2)	4.5 (1.1–6.0)
SAA elevation > 6.4, *n* (%)	5 (35.7%)	5 (35.7%)	3 (21.4%)
**Complement factors (g/L), median (25th−75th percentile)**			
C3	1.136 (1.086–1.302)^*^	1.245 (1.102–1.336)^†^	1.001 (0.858–1.190)
C4	0.193 (0.170–0.243)^*^	0.216 (0.146–0.245)^†^	0.158 (0.140–0.197)
Hypocomplementemia, *n* (%)	0 (0.0)^*^	0 (0.0)^†^	4 (28.6)
IL-6 (pg/mL), median (25th−75th percentile)	2.5 (1.8–4.0)^*^	3.1 (1.6–4.7)^†^	1.4 (1.4–1.7)
Elevated IL-6, *n* (%)	0 (0.0)	0 (0.0)	0 (0.0)
Positive ANA titer ≥ 1:80, *n* (%)	5 (35.7)	4 (28.6)	4 (28.6)
ENA (U/mL), median (25th−75th percentile)	0.0 (0.0–0.1)	0.1 (0.0–0.1)	0.1 (0.0–0.2)
Elevated ENA, *n* (%)	0 (0.0)	0 (0.0)	0 (0.0)
**Antiphospholipid antibodies**			
Lupus anticoagulant (sec), median (25th−75th percentile)	32.8 (31.2–38.5)	35.3 (33.8–37.4)	33.7 (31.6–35.3)
Elevated lupus anticoagulant, *n* (%)	0 (0.0)	1 (7.1)	0 (0.0)
Total level of cardiolipin antibodies including IgA, IgG, IgM cardiolipin antibodies (U/mL), median (25th−75th percentile)	1.7 (0.1–3.9)	3.1 (2.5–4.4)^†^	0.5 (0.3–1.1)
Elevated cardiolipin antibodies, *n* (%)	2 (14.3)	0 (0.0)	0 (0.0)
Total level of β-2-glycoprotein antibodies including IgA, IgG, IgM β-2-glycoprotein antibodies (U/mL), median (25th−75th percentile)	2.2 (1.6–2.7)^*^	2.3 (1.9–2.4)^†^	1.6 (1.5–1.8)
Elevated β-2-glycoprotein antibodies, *n* (%)	1 (7.1)	0 (0.0)	0 (0.0)
Any elevated antiphospholipid antibody, *n* (%)	2 (14.3)	1 (7.1)	0 (0.0)
**ANCA**			
MPO-ANCA (U/mL), median (25th−75th percentile)	0.8 (0.6–1.1)	0.8 (0.8–1.0)^†^	1.2 (0.8–1.3)
Elevated MPO-ANCA, *n* (%)	0 (0.0)	0 (0.0)	0 (0.0)
PR3-ANCA (U/mL), median (25th−75th percentile)	0.4 (0.4–1.9)^‡^	0.4 (0.4–0.4)^†^	0.5 (0.4–2.4)
Elevated PR3-ANCA, *n* (%)	0 (0.0)	0 (0.0)	0 (0.0)
Positive c-ANCA titer ≥ 1:80, *n* (%)	0 (0.0)	1 (7.1)	0 (0.0)
Positive p-ANCA titer ≥ 1:80, *n* (%)	0 (0.0)	0 (0.0)	0 (0.0)
Positive x-ANCA titer ≥ 1:80, *n* (%)	0 (0.0)	0 (0.0)	0 (0.0)
ACP antibodies (U/mL), median (25th−75th percentile)	0.9 (0.5–1.1)	0.8 (0.5–1.1)	0.9 (0.6–1.1)
Elevated ACP antibodies, *n* (%)	0 (0.0)	0 (0.0)	0 (0.0)
Rheumatoid factor (U/mL), median (25th−75th percentile)	0 (0–7)	0 (0–1)	0 (0–8)
Elevated rheumatoid factor, *n* (%)	0 (0.0)	0 (0.0)	1 (7.1)
Positive cytoplasmic antibodies, *n* (%)	1 (7.1)	1 (7.1)	0 (0.0)
**Ig (g/L), median (25th−75th percentile)**			
IgA	1.88 (1.07–2.82)	1.77 (1.60–2.42)	1.52 (1.18–2.00)
<0.7, *n* (%)	1 (7.1)	1 (7.1)	0 (0.0)
> 4, *n* (%)	1 (7.1)	1 (7.1)	1 (7.1)
IgG	10.55 (8.47–12.10)	9.13 (8.25–10.30)	9.61 (9.13–10.80)
<7, *n* (%)	1 (7.1)	1 (7.1)	0 (0.0)
> 16, *n* (%)	0 (0.0)	0 (0.0)	0 (0.0)
IgM	1.03 (0.72–1.24)^‡^	0.55 (0.49–0.76)^†^	0.83 (0.57–1.04)
<0.4, *n* (%)	0 (0.0)	1 (7.1)	0 (0.0)
> 2.3, *n* (%)	0 (0.0)	1 (7.1)	0 (0.0)
IgG_1_	6.44 (5.02–8.14)	6.11 (5.33–7.72)	5.99 (5.41–7.45)
<4.05, *n* (%)	0 (0.0)	1 (7.1)	0 (0.0)
> 10.11, *n* (%)	0 (0.0)	0 (0.0)	1 (7.1)
IgG_2_	3.00 (2.12–4.31)^‡^	2.27 (1.77–2.72)^†^	3.46 (2.5–3.88)
<1.69, *n* (%)	0 (0.0)	2 (14.3)	0 (0.0)
> 7.86, *n* (%)	0 (0.0)	0 (0.0)	0 (0.0)
IgG_3_	0.35 (0.17–0.41)	0.28 (0.25–0.43)	0.32 (0.27–0.41)
<0.11, *n* (%)	2 (14.3)	0 (0.0)	0 (0.0)
> 0.85, *n* (%)	0 (0.0)	0 (0.0)	0 (0.0)
IgG_4_	0.41 (0.13–1.04)	0.23 (0.11–1.02)	0.45 (0.27–0.54)
<0.03, *n* (%)	2 (14.3)	0 (0.0)	0 (0.0)
>2.01, *n* (%)	1 (7.1)	0 (0.0)	0 (0.0)
Any Ig elevation, *n* (%)	2 (14.3)	2 (14.3)	2 (14.3)
Any Ig decrease, *n* (%)	5 (35.7)^*^	4 (28.6)^†^	0 (0.0)
Total number of pathologically altered inflammatory conditions, median (25th−75th percentile)	2 (1–3)^*^	2 (1–2)	1 (0–2)

*ACP, anti-citrullinated protein; ANA, antinuclear antibodies; ANCA, anti-neutrophil cytoplasmic antibodies; ASCVD, atherosclerotic cardiovascular diseases; CRP, C-reactive protein; ENA, extractable nuclear antigen; ESR, erythrocyte sedimentation rate; Ig, immunoglobulin; IL-6, interleukin 6; SAA, serum amyloid A; WBC, white blood cells*.

* *p < 0.05 between group with previous COVID-19 and healthy controls*.

† *p < 0.05 between group with ASCVD and healthy controls*.

‡ *p < 0.05 between group with previous COVID-19 and group with ASCVD*.

### Microvascular Changes

Capillary ramifications, loss, caliber variability, and elongations were more frequently observed in post-COVID-19 patients compared to patients with ASCVD (*p* = 0.015; *p* = 0.034; *p* = 0.047; *p* = 0.020, respectively) and capillary ramifications, loss, caliber variability and bushy capillaries were more frequently compared to healthy controls (*p* = 0.015; *p* = 0.034; *p* = 0.003; *p* = 0.014, respectively). Using a semi-quantitative rating scale, significantly higher score values were achieved for capillary ramifications, capillary loss and elongated capillaries in the group with previous COVID-19 compared to the group with ASCVD (*p* = 0.016; *p* = 0.035; *p* = 0.028, respectively). Higher score values were also observed for capillary ramifications, loss, caliber variability, elongation and bushy capillaries compared to healthy controls (*p* = 0.016; *p* = 0.035; *p* = 0.003; *p* = 0.028; *p* = 0.018, respectively). Total MES was higher in post-COVID-19 patients compared to patients with ASCVD (*p* = 0.048) and to healthy controls (*p* = 0.040) (Table 5).

**Table 5:** Capillary changes.

	**COVID-19 (*n* = 14)**	**ASCVD (*n* = 14)**	**Controls (*n* = 14)**
**Microhemorrhages**			
*n*, (%)	8 (57.1)	6 (42.9)	3 (21.4)
Points, median (25th−75th percentile)	0.125 (0.000–0.250)	0.000 (0.000–0.125)	0.000 (0.000–0.031)
**Capillary edema**			
*n*, (%)	0 (0.0)	0 (0.0)	0 (0.0)
Points, median (25th−75th percentile)	0.000 (0.000–0.000)	0.000 (0.000–0.000)	0.000 (0.000–0.000)
**Capillary ramifications**			
*n*, (%)	5 (35.7)^*^^‡^	0 (0.0)	0 (0.0)
Points, median (25th−75th percentile)	0.000 (0.000–0.125)^*^^‡^	0.000 (0.000–0.000)	0.000 (0.000–0.000)
**Bushy capillaries**			
*n*, (%)	7 (50.0)^*^	3 (21.4)	1 (7.1)
Points, median (25th−75th percentile)	0.063 (0.000–0.156)^*^	0.000 (0.000–0.031)	0.000 (0.000–0.000)
**Capillary loss**			
*n*, (%)	4 (28.6)^*^^‡^	0 (0.0)	0 (0.0)
Points, median (25th−75th percentile)	0.000 (0.000–0.469)^*^^‡^	0.000 (0.000–0.000)	0.000 (0.000–0.000)
**Giant capillaries (≥50 μm)**			
*n*, (%)	0 (0.0)	0 (0.0)	0 (0.0)
Points, median (25th−75th percentile)	0.000 (0.000–0.000)	0.000 (0.000–0.000)	0.000 (0.000–0.000)
**Capillary ectasia (≥25 μm)**			
*n*, (%)	1 (7.1)	2 (14.3)	0 (0.0)
Points, median (25th−75th percentile)	0.000 (0.000–0.000)	0.000 (0.000–0.000)	0.000 (0.000–0.000)
**Tortuous capillaries**			
*n*, (%)	12 (85.7)	8 (57.1)	9 (64.3)
Points, median (25th−75th percentile)	0.500 (0.125–1.375)	0.375 (0.000–1.063)	0.125 (0.000–0.531)
**Capillary caliber variability**			
*n*, (%)	7 (50.0)^*^^‡^	2 (14.3)	0 (0.0)
Points, median (25th−75th percentile)	0.063 (0.000–0.281)^*^	0.000 (0.000–0.000)	0.000 (0.000–0.000)
**Elongated capillaries**			
*n*, (%)	8 (57.1)^‡^	2 (14.3)	3 (21.4)
Points, median (25th−75th percentile)	0.125 (0.000–0.469)^*^^‡^	0.000 (0.000–0.000)	0.000 (0.000–0.031)
**Capillary thrombosis**			
*n*, (%)	0 (0.0)	1 (7.1)	0 (0.0)
Points, median (25th−75th percentile)	0.000 (0.000–0.000)	0.000 (0.000–0.000)	0.000 (0.000–0.000)
**Disorganization of microvascular array**			
*n*, (%)	12 (85.7)	8 (57.1)	8 (57.1)
Points, median (25th−75th percentile)	1.063 (0.250–2.156)	0.375 (0.000–1.313)	0.313 (0.000–1.125)
Early pattern, *n* (%)	0 (0.0)	0 (0.0)	0 (0.0)
Active pattern, *n* (%)	0 (0.0)	0 (0.0)	0 (0.0)
Late pattern, *n* (%)	0 (0.0)	0 (0.0)	0 (0.0)
CSURI (points)	–^§^	–^§^	–^§^
MES (points), median (25th−75th percentile)	1.630 (0.250–2.438)^*^^‡^	0.375 (0.000–1.313)	0.315 (0.00–1.125)

*ASCVD, atherosclerotic cardiovascular diseases; CSURI, capillaroscopic skin ulcer risk index; MES, microangiopathy evolution score*.

* *p < 0.05 between group with previous COVID-19 and healthy controls*.

† *p < 0.05 between group with ASCVD and healthy controls*.

‡ *p < 0.05 between group with previous COVID-19 and group with ASCVD*.

§ *not detectable*.

## Discussion

We could demonstrate substantial differences of selected pathways contributing to endothelial dysfunction in patients 6 months after SARS-CoV-2 infection. Although no differences were observed for markers of vascular reactivity, post-COVID-19 patients had an increased arterial stiffness, distinct alterations of the arginine and kynurenine metabolism, and higher values of IMT, vWF, homocysteine, and CD31+/CD42b– EMP compared to healthy controls. Additionally, many of the respective parameters, including also FMD and NMD, were altered to an extent comparable with the values of patients with clinically relevant ASCVD; 78.6% of those had a prior endovascular intervention. Furthermore, capillary changes have been observed more frequently in post-COVID-19 patients compared to healthy controls and the group of ASCVD including also a higher MES. Changes for most of the respective parameters have previously been described in patients mainly with acute COVID-19 while data about persistent changes after suffered COVID-19 are very limited (9, 35–40).

Pathophysiological mechanisms contributing to endothelial dysfunction in COVID-19 are largely unknown. Direct and indirect endothelial damage due to SARS-CoV-2 by binding to the angiotensin-converting-enzyme-2 receptor and by acute systemic inflammation have been proposed (4, 41). While direct infection of endothelial cells by SARS-CoV-2 may be unlikely, as there is lacking evidence of expression of angiotensin-converting-enzyme-2 receptor on human endothelial cells, indirect endothelial damage by release of inflammatory mediators may affect several pathways contributing to endothelial dysfunction, including nitric oxide or kynurenine metabolism, resulting subsequently in impaired FMD and increased arterial stiffness (22, 42–45). Our findings support the hypothesis of indirect endothelial damage caused by systemic inflammation. On the one hand, post-COVID-19 patients revealed numerous altered parameters of endothelial dysfunction, and subclinical inflammation expressed by elevated levels of CRP, ESR, and IL-6 as well as by a higher total number of pathologically altered inflammatory conditions (17, 25, 26). Respective inflammatory changes of post-COVID-19 patients were again similar to those observed in patients with ASCVD. Furthermore, although FMD and NMD did not differ between the three cohorts, post-COVID-19 patients had similar values of FMD and NMD compared to patients with ASCVD and also the number of post-COVID-19 with pathologic FMD and NMD values according to proposed reference values were similar compared to patients with ASCVD (25, 26). Interestingly, FMD and NMD values of our healthy control cohort were also comparable to FMD and NMD values of post-COVID-19 and ASCVD patients, which may be attributed to other subject-related factors influencing vascular reactivity, like smoking, physical activity, mental stress, alcohol intake or hormonal changes during physiological menstrual cycle (13). The association between inflammation and atherosclerosis is well-established and it may be possible that persisting changes of inflammatory parameters caused by SARS-CoV-2 may affect endothelial cells similarly (46). On the other hand, the occurrence of persisting capillary changes in post-COVID-19 patients also suggests an interaction *via* inflammation and immunological pathways. Capillary changes have mainly been described in autoimmune disorders, especially in systemic sclerosis (32–34). Interestingly, post-COVID-19 patients had a higher prevalence of capillary ramifications and capillary loss, which are typically seen in long-lasting systemic sclerosis, while no capillary pattern suggestive for systemic sclerosis were observed. Compared to the study of Natalello et al. (9), we could observe less capillary edema, thrombosis and ectasia but higher rates of capillary ramifications, bushy capillaries and capillary loss. Additionally, higher rates of capillary caliber variability and elongations were observed and higher scores using semi-quantitative rating scale of respective capillary changes and total MES were found in post-COVID-19 patients. As connective tissues diseases or vasculitides were an exclusion criterion, it can be assumed that SARS-CoV-2 affects substantially and persistently microvasculature. Finally, endothelial damage caused by SARS-CoV-2 *per se* without interaction *via* inflammatory pathways may also be a potential pathophysiologic explanation for COVID-19-associated endothelial dysfunction and vasculopathy. Associations between EMP and parameters of the arginine metabolism to other viruses, like parvovirus B19 or human immunodeficiency virus, have previously been described (47, 48). Furthermore, ADMA and CD31+/CD42b– EMP have been associated with capillary changes in systemic sclerosis (49, 50). Therefore, direct but yet unknown interactions of SARS-CoV-2 to nitric oxide metabolism or endothelial homeostasis may also contribute to the persistent endothelial dysfunction and vasculopathy observed in our study.

Limitations of our study are that this study included a limited number of patients and the fact that we did not measure the above-named parameters before, during and after SARS-CoV-2 infection to evaluate potential changes. Therefore, it can be only hypothesized that persisting endothelial damage is caused directly or indirectly due to COVID-19. However, measuring endothelial function in people before COVID-19 is challenging, given that one would need to assess a large number of people to ascertain that a subgroup will have a SARS-CoV-2 infection. Additionally, while no post-COVID-19 patient had any preexisting ASCVD, most of them had at least one atherosclerotic cardiovascular risk factor. Although those cardiovascular risk factors were not significantly different or overrepresented in post-COVID-19 patients, a potential bias affecting the results on endothelial dysfunction due to present cardiovascular risk factors cannot be definitely excluded. Furthermore, the large age difference between the control group and the two patient groups need to be mentioned which may affect several measured parameters. However, the aim of this study was to compare parameters of endothelial dysfunction and inflammation in post-COVID-19 patients between a healthy group without suspected alterations and a group of patients with suspected altered parameters to rank the potential influence of COVID-19 on endothelial dysfunction and inflammatory vasculopathy. Therefore, young, healthy and sex-matched controls were used instead of age-matched controls.

Strengths of our study are that all parameters were measured together in one study cohort with balanced sex and age distribution and a quite homogenous COVID-19 phenotype. Another strength of our study is that we included a healthy control and a sex- and age-matched ASCVD control group to discriminate the impact of COVID-19 on endothelial dysfunction and inflammatory vasculopathy, which has not been done before in studies investigating endothelial function in people who had COVID-19. In previous studies, different COVID-19 phenotypes and COVID-19 subjects with several cardiovascular comorbidities were commonly included (9, 34–39). A further strength is that all controls had no proven recent or prior SARS-CoV-2 infection at study measurement.

In conclusion, COVID-19 may contribute to enhanced endothelial dysfunction and disturbed vascular homeostasis *via* influence of EMP, inflammatory pathways as well as of arginine, kynurenine and homocysteine metabolism. Thus, changes of arterial stiffness, vascular reactivity and microvasculature may be promoted after SARS-CoV-2 infection. Further studies are needed to elucidate the underlying pathways of COVID-19 associated endothelial dysfunction and to clarify if those vascular changes are long-lasting and if COVID-19 may be even a potential risk factor for the development of atherosclerotic or inflammatory vascular diseases.

## Data Availability Statement

The original contributions presented in the study are included in the article/supplementary material, further inquiries can be directed to the corresponding author/s.

## Ethics Statement

The studies involving human participants were reviewed and approved by Institutional Review Board of the Medical University Graz. The patients/participants provided their written informed consent to participate in this study.

## Author Contributions

PJ contributed to conception of the manuscript, subject recruitment, data acquisition, data interpretation, and writing of the manuscript. PG contributed to conception of the manuscript and data acquisition. VM and HSo contributed to subject recruitment. AA contributed to statistical analysis. AM, HSt, RR, MS, UD, and HK contributed to data analysis and interpretation. KE contributed to data acquisition. MB contributed to conception and supervision of the manuscript. All authors revised the manuscript and gave their final approval of the manuscript version to be published.

## Conflict of Interest

The authors declare that the research was conducted in the absence of any commercial or financial relationships that could be construed as a potential conflict of interest.

## Publisher's Note

All claims expressed in this article are solely those of the authors and do not necessarily represent those of their affiliated organizations, or those of the publisher, the editors and the reviewers. Any product that may be evaluated in this article, or claim that may be made by its manufacturer, is not guaranteed or endorsed by the publisher.

## Abbreviations

ACP: anti-citrullinated protein
ADMA: asymmetric dimethylarginine
AECA: antiendothelial cell antibodies
ANA: antinuclear antibodies
ANCA: anti-neutrophil cytoplasmic antibodies
ASCVD: atherosclerotic cardiovascular diseases
CRP: C-reactive protein
CSURI: capillaroscopic skin ulcer risk index
ELISA: enzyme-linked immunosorbent assay
EMP: endothelial microparticles
ENA: extractable nuclear antigen
ESR: erythrocyte sedimentation rate
FMD: flow-mediated dilation
GAB: global arginine bioavailability
Ig: immunoglobulin
IL-6: interleukin 6
IMT: intima-media thickness
LEAD: lower extremity arterial disease
MES: microangiopathy evolution score
NMD: nitroglycerine-related dilation
NVC: nailfold video capillaroscopy
PCR: polymerase chain reaction
PWV: pulse-wave velocity
SAA: serum amyloid A
SARS-CoV-2: severe acute respiratory syndrome coronavirus 2
SD: standard deviation
SDMA: symmetric dimethylarginine
UEAD: upper extremity arterial disease
vWF: von Willebrand factor.
